# KDT501, a Derivative from Hops, Normalizes Glucose Metabolism and Body Weight in Rodent Models of Diabetes

**DOI:** 10.1371/journal.pone.0087848

**Published:** 2014-01-30

**Authors:** Veera R. Konda, Anuradha Desai, Gary Darland, Neile Grayson, Jeffrey S. Bland

**Affiliations:** KinDex Therapeutics, LLC, Seattle, Washington, United States of America; National Institute of Nutrition, India

## Abstract

**Aims/Hypothesis:**

We developed KDT501, a novel substituted 1,3-cyclopentadione chemically derived from hop extracts, and evaluated it in various *in vitro* and *in vivo* models of diabetes and insulin sensitivity.

**Methods:**

KDT501 was evaluated for anti-inflammatory effects in monocyte/macrophage cells; agonistic activity for peroxisome proliferator-activated receptors (PPAR); lipogenesis and gene expression profile in human subcutaneous adipocytes. Body composition, glucose, insulin sensitivity, and lipids were assessed in diet-induced obesity (DIO) mice and Zucker Diabetic Fatty (ZDF) rats after oral administration.

**Results:**

KDT501 mediated lipogenesis in 3T3L1 and human subcutaneous adipocytes; however, the gene expression profile of KDT501 differed from that of the full PPARγ agonist rosiglitazone, suggesting that KDT501 has pleiotropic biological activities. In addition, KDT501 showed only modest, partial PPARγ agonist activity and exhibited anti-inflammatory effects in monocytes/macrophages that were not observed with rosiglitazone. In a DIO mouse model, oral administration of KDT501 significantly reduced fed blood glucose, glucose/insulin AUC following an oral glucose bolus, and body fat. In ZDF rats, oral administration of KDT501 significantly reduced fed glucose, fasting plasma glucose, and glucose AUC after an oral glucose bolus. Significant, dose-dependent reductions of plasma hemoglobin A1c, weight gain, total cholesterol, and triglycerides were also observed in animals receiving KDT501.

**Conclusion:**

These results indicate that KDT501 produces a unique anti-diabetic profile that is distinct in its spectrum of pharmacological effects and biological mechanism from both metformin and pioglitazone. KDT501 may thus constitute a novel therapeutic agent for the treatment of Type 2 diabetes and associated conditions.

## Introduction

The molecular link between inflammation, obesity, and insulin resistance is incompletely understood; however, multiple lines of evidence suggest that inflammation plays a major role in insulin resistance. A number of groups have investigated the link between inflammation, obesity and insulin resistance [1], [2]. Several studies have recently reported that obesity leads to the infiltration of macrophages and immune cells into adipose tissue, activating an inflammatory response that leads to insulin resistance [3], [4]. In lean mice, adipose tissue macrophages having an alternatively activated (M2) phenotype are less inflammatory and may actually afford protection, whereas macrophages in adipose tissue of obese mice have a pro-inflammatory, classical (M1) phenotype [5], [6]. These studies suggest an important link between macrophage-mediated inflammation in obesity-related insulin resistance.

PPAR-gamma (PPARγ) is the primary target of the drug class of thiazolidinediones (TZDs), which are used to treat diabetes mellitus and other diseases featuring insulin resistance. It has been shown that activation of PPARγ increases adipocyte differentiation, lipid metabolism, insulin sensitivity, and glucose homeostasis [7]. Besides their beneficial role as insulin-sensitizing agents in Type 2 diabetes mellitus (T2D), TZD drugs such as rosiglitazone and pioglitazone are known to produce adverse effects including weight gain, plasma volume expansion, edema, and cardiac hypertrophy in preclinical species [8]. The edema that can accompany the use of TZDs has been linked to congestive heart failure in humans [9].

In contrast to full PPARγ agonists, partial PPARγ agonists mitigate insulin resistance and hyperglycemia in rodent models of obesity without increasing adiposity and cardiac weight [10]. Furthermore, structurally distinct, non-TZD PPARγ modulators exhibit anti-diabetic properties with improved therapeutic indices with regard to excessive fat and cardiomegaly compared to rosiglitazone [11]–[14] in rodent models. Recent efforts to develop safer anti-diabetic molecules with partial PPARγ activity include the identification and validation of a partial PPARγ agonist of natural origin [15].

Several studies have reported potential anti-inflammatory effects of PPARγ ligands on monocytes/macrophages. However, it has been demonstrated that PPARγ activation is not essential for PPARγ ligands to exert anti-inflammatory effects in macrophages. For example, in a RAW264.7 cell model [16], the non-TZD PPARγ agonist L-796,449 displayed anti-inflammatory properties even though PPARγ expression was minimal or absent. As another example, abscisic acid increased PPARγ activity independent of its ligand binding mechanism [17] and reduced obesity-related inflammation and promoted glucose tolerance in db/db mice. [18]. These studies suggest that many partial or non-TZD PPARγ agonists produce anti-inflammatory activity independent of PPARγactivation or binding.

Extracts from hops (*Humulus lupulus*) have been widely used as flavoring agents in brewing. Previously, it was reported that mixtures of hop extracts show anti-inflammatory activity by inhibiting the NF-κB signaling pathway [19]-[21], exhibit anti-diabetic effects [22], [23], and reduce systemic inflammation in high fat diet-induced obesity (DIO) mice [24]. We developed KDT501, a novel, stereochemically pure substituted 1,3-cyclopentadione, chemically derived from hop extracts and assigned the correct stereochemistry to this molecule and, for the first time, to other humulone pharmacophores [25]. In this study, we evaluated KDT501 in various *in vitro* and *in vivo* models of diabetes and insulin sensitivity.

## Materials

Lipopolysaccharide (LPS), TNFα and telmisartan were purchased from Sigma (St. Louis, MO).

Milliplex MAP human cytokine/chemokine kit and adipogenesis assay kits were purchased from Millipore (Billerica, MA). 3T3-L1 cells, THP-1 cells and FBS were purchased from ATCC (Manassas, VA). RPMI-1640 medium was purchased from Hyclone (Logan, UT). Penicillin-streptomycin solution was purchased from MediaTech (Manassas, VA). PPARα, PPARγ and PPARδ assay kits, rosiglitazone, GW590735 and GW0742 were from Indigo Biosciences (State College, PA). DMEM/F12 medium was purchased from Invitrogen (Carlsbad, CA). Human subcutaneous primary adipocytes and cell culture reagents were purchased from Lonza (Walkersville, MD). All other reagents were analytical grade.

## Methods

### Cerep Bioprint assays

KDT501 was screened over 150 (Cerep BioPrint Panel, Poitiers, FR) biological targets, binding and enzymatic assays, as well as *in vitro* screens focused on ADME related issues. The protocols and target list are available at (http://www.cerep.fr/cerep/users/pages/catalog/profiles/DetailProfile.asp?profile=2130).

### PPARγ reporter assays

PPAR assay kits from Indigo Biosciences and supplemented protocols were used in this study. Briefly, the reporter cells were treated in triplicates with various concentrations of KDT501 (25–0.78 µM). Telmisartan (10–0.625 µM), GW590735 (10–0.03 µM), and GW0742 (1–0.01 µM) were used as positive controls for PPARγ, PPARα and PPARδ respectively. Rosiglitazone (1–0.031 µM) was used in all assays to confirm specificity to PPARγ. DMSO (0.1%) was used as a negative control. Cells were incubated for 20 hours as per the protocol and read using a luminometer (Victor2, Perkin Elmer). Average relative light unit (RLU) values from blank wells were subtracted from all the samples. The final data were compared with DMSO control and statistical analyses were performed using GraphPad Prism.

### THP-1 cells

THP-1 cells were maintained in RPMI1640 in the presence of 10% serum according to manufacturer's instructions as described earlier [20]. The cells were pre-incubated with various concentrations of test compounds (KDT501, rosiglitazone, telmisartan, DHA or PGJ2) in the presence of 1% serum medium for 1 hour, then stimulated with TNF-α (10 ng/ml) or LPS (1 µg/ml) overnight (16–20 hours. Cytokines were assayed using a Milliplex MAP human cytokine/chemokine kit and Luminex 100™ IS. Data were analyzed using a five-parameter logistic method and represented as Mean ±SD of four independent samples.

### Lipogenesis

Murine 3T3-L1 preadipocytes were maintained in DMEM supplemented with 10% FBS as described earlier [26]. Cells were seeded at an initial density of approximately 1.5×10^6^ cells in 24-well plates. After confluence, cells were treated with KDT501 (25, 12.5, 6.25, and 3.25 µM), rosiglitazone (10 µM), or DMSO on day 0. Cells were differentiated in differentiation medium consisting of 10% FBS/DMEM, 0.5 mM methylisobutylxanthine, 0.5 µM dexamethasone, and 10 µg/mL insulin. After two days, the medium was changed to progression medium consisting of 10 µg/mL insulin in 10% FBS/DMEM followed by 2 more days in maintenance medium consisting of 10% FBS/DMEM. KDT501 or rosiglitazone were included throughout the maturation phase (day 6/day 7). Human subcutaneous primary adipocytes were maintained as described for 3T3L1 adipocytes and treated with rosiglitazone (1 µM), KDT501 (10 µM), telmisartan (10 µM), DHA (10 µM) or PGJ2 (10 µM) for 10 days.

### Oil O-red staining

Intracellular lipid was quantified with Oil O-Red staining using an adipogenesis assay kit from (Millipore, Billerica, MA). The medium was carefully discarded and washed twice with PBS. 300 µL Of Oil O-Red solution were added and incubated for 15 minutes at room temperature. The cells were washed, and bound dye was extracted and transferred to a 96 well plate. Absorbance was measured at 530 nm using a plate reader (Thermo Electron Corp.). Fold change relative to DMSO control was calculated from four independent wells for each condition. Data represent Mean ± SD.

### Gene Expression analysis

Human subcutaneous primary adipocytes were maintained as described earlier. Cells were seeded at an initial density of approximately 2×10^6^ cells in 6-well plates with PGM-2 medium (2 mL medium/well) and allowed to grow for 24 hr to confluence. Cells were treated with test compounds KDT501 (25 µM), rosiglitazone (1 µM), telmisartan (10 µM), or DMSO in adipocyte differentiating medium. The differentiation medium consisted of indomethacin, methylisobutylxanthine, dexamethasone, and insulin from PGM-2 single aliquots. After 5 days, the cells were washed in PBS, frozen in liquid nitrogen and shipped to Expression Analysis (Durham, NC) in dry ice. Three independent plates were used for each condition.

RNA was extracted using the miRNeasy Mini Kit (Qiagen) as described by the manufacturer. RNA was isolated and expression profiling was performed using the Ambion WT Expression Kit (Life Technologies). Labeled target, which was hybridized to GeneChip Human Gene 1.0 ST Arrays (Affymetrix), was prepared essentially as described by the manufacturer. This cDNA was then used as a template for *in vitro* transcription using T7 RNA Polymerase to produce anti-sense RNA, which was then converted into single-stranded sense cDNA that was fragmented and labeled with biotin prior to hybridization to the GeneChip Arrays. Stained arrays were imaged using a Scanner 3000 (Affymetrix) and probe set analysis results were generated with Expression Console (Affymetrix) using the robust multi-array algorithm.

Gene Ontology (GO): Gene expression fold-change was calculated for test compounds ≥ absolute (log2) relative to control and with a p-value <0.05 and PADE value <15. The Bonferroni adjusted p-value (<0.05) was used for ranking and is shown as “E-Value”. The GO terms for KDT501 (Probe Set 24), telmisartan (Probe Set 8) and rosiglitazone (Probe Set 74) denote direct biological pathways.

### Animal Care and Use Statement

All procedures were in compliance with the U.S. Department of Agriculture's (USDA) Animal Welfare Act (9 CFR Parts 1, 2, and 3) and other appropriate agencies. All animal studies were approved by the Institutional Animal Care and Use Committee of Covance Laboratories (Greenfield, IN).

### DIO Mice

C57Bl6/J male mice were 15 weeks old at the start of the experiment. In general, DIO models are initiated at an early age to evaluate effects on obesity. However, older, overweight mice were used in this study to represent middle-aged human subjects for the assessment of metabolic parameters associated with weight loss/diabetes. The mice were housed individually in cages at 72±4°F with a relative humidity of 30–70% with 12 hour light- dark cycle. High fat diet (40% Cal) TD95217 (Teklad Custom Research Diet) was used in this study. The test compounds were administered orally twice per day at 12 hour intervals (10 mL/kg) in 0.5% methylcellulose and 0.2% Tween 80 (w/v). Mice were randomly allocated on the basis of body weight to seven groups (N = 10/group), with five animals for the oral glucose tolerance test (OGTT) and 5 animals for the insulin tolerance test. KDT501 (25, 50, 100 and 200 mg/kg, calculated as the free acid), pioglitazone (30 mg/kg) and metformin (200 mg/kg) were given twice per day. Body weight and food consumption were determined weekly.

After 30 days, the mice were fasted overnight, blood was drawn through the tail vein and baseline blood glucose was measured using a glucose analyzer. One hour later, mice were given an oral gavage of glucose solution in water (4 ml/kg, 2 g/kg) and blood samples were taken at 15, 30, 60, and 120 min to determine plasma glucose and insulin levels. Five days before the start of the experiment (t = −5 days) and again at the end of the experiment (t = 28 days), body fat was determined using QNMR. With the exception of the group treated with 200 mg/kg KDT501, 10 animals/group were used in the determinations. Insulin and HbA1c glucose levels were determined on days 1, 15 and 29.

### Zucker Diabetic Fatty (ZDF) Rats

Male ZDF rats were obtained from Charles River Laboratories at approximately 7 weeks of age. Rats were maintained ad lib on Purina #5008 diet, delivering approximately 17% of calories from fat. Animals with fasting glucose between 150–350 mg/dL were randomized based on glucose and body weight. Test articles were prepared in 0.5% (w/v) methylcellulose + 0.2% (w/v) Tween 80 and given by oral gavage twice daily. Rats (10/group, 6 treatment groups) were administered KDT501 (100, 150, or 200 mg/kg), metformin (200 mg/kg), pioglitazone (30 mg/kg) or vehicle control for up to 32 days. Body weights and food intake were determined weekly.

Blood was collected via tail bleed and whole blood glucose was measured on days 1, 8, 15 and 29. Blood was collected in 5 animals on days 15 and 29 for determination of glucose, insulin, lipids, hematocrit, and HbA1c. Animals were fasted overnight and OGTT was conducted on days 31 and 32 for determination of glucose and insulin levels. A second ZDF rat study was conducted and the resulting data on total cholesterol are presented.

#### Statistical Methods for animal studies

Statistical calculations were performed (JMP version, SAS Institute, Cary, NC). One-way ANOVA with Dunnett's test for *post hoc* determinations were used to compare vehicle treated control. Data were expressed as mean ± SEM.

## Results

### Screening for Biological targets

As a first step in our investigation, KDT501 was screened in Cerep's BioPrint Panel to identify its biological targets. Only three binding targets were identified: the ion channel, SCN2a, the type II angiotensin receptor, AGTR2, and PPARγ, with IC_50_'s of 19, 14 and 8.4 µM, respectively. Since we were aware of the pleiotropic nature of hop components, we then embarked on a series of analyses to distinguish KDT501 at a molecular level from traditional glitazone PPARγ agonists.

### Effect of KDT501 on PPAR reporter activity

**Figure 1:**
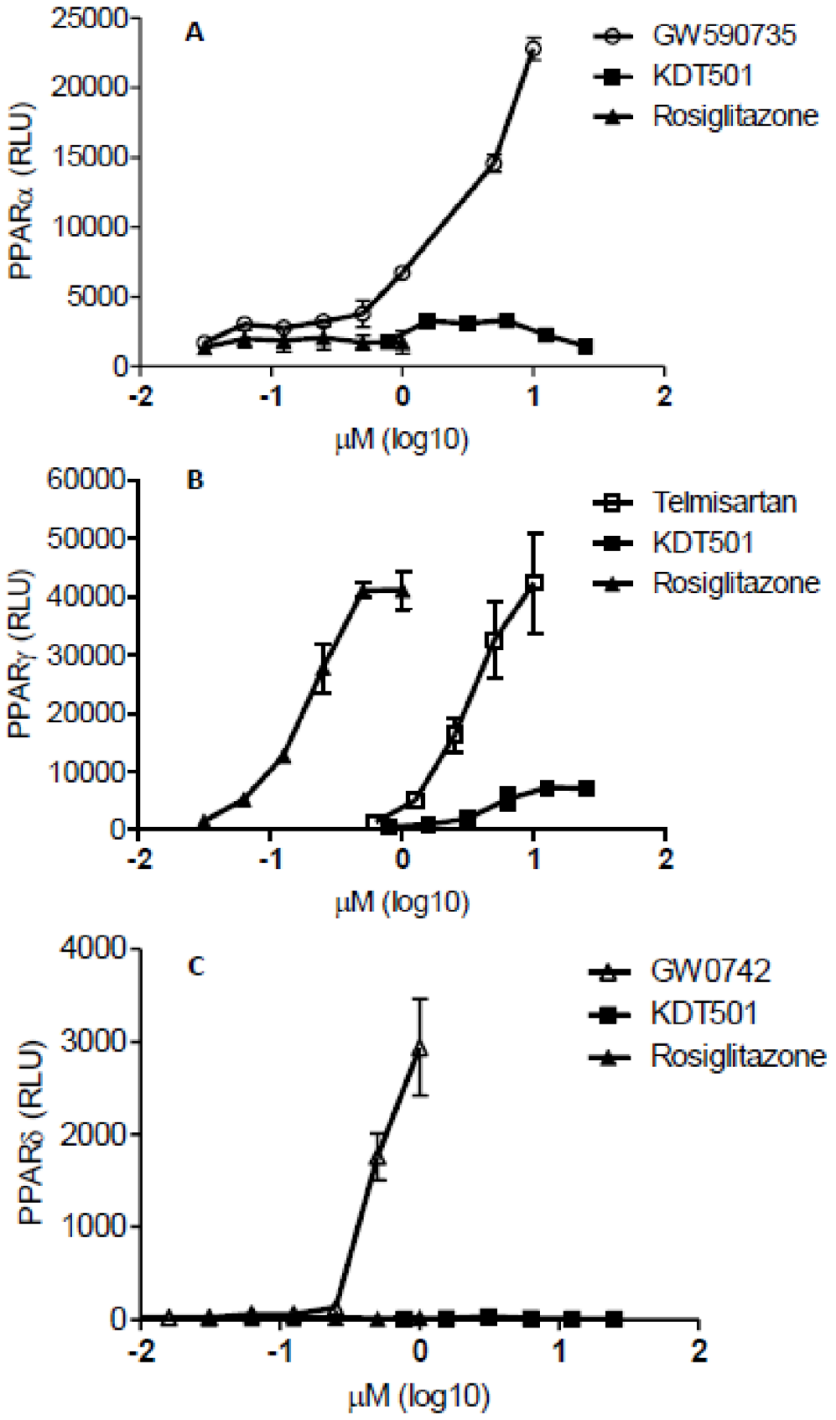
KDT501 agonistic effect on PPAR reporter activity. PPARα reporter cells were treated with various concentrations of GW590735 (round circles), rosiglitazone (black triangles) and KDT501 (black squares) (A). PPARγ reporter cells were treated with various concentrations of rosiglitazone, telmisartan (white squares), and KDT501 (B). PPARδ reporter cells were treated with GW0742 (white triangles), rosiglitazone and KDT501(C). Cells were treated for 20 hr and relative luminescence units (RLU) were measured as described in the methods. Agonist concentrations were transformed to log10 µM. Agonist dose- responses were plotted by non-linear regression. Data represent Mean± SD.

High concentrations of KDT501 increased PPARγ reporter activity, but had no effect on PPARα or PPARδ reporter activity (Figure 1), suggesting that KDT501 is a specific agonist of PPARγ. Similar to rosiglitazone, telmisartan, a known PPARγ agonist, also increased PPARγ activity (Fig 1B) with lower potency (EC_50_ = 13.4 µM) compared to rosiglitazone (EC_50_ = 0.42 µM). On the other hand, KDT501 (EC_50_ = 14.0 µM) exhibited both weak and partial PPARγ activity, reaching only 29±7% (n = 3, Mean ±SEM) of rosiglitazone's maximum activation.

### Anti-inflammatory properties of KDT501 in LPS-activated THP-1 cells

**Figure 2:**
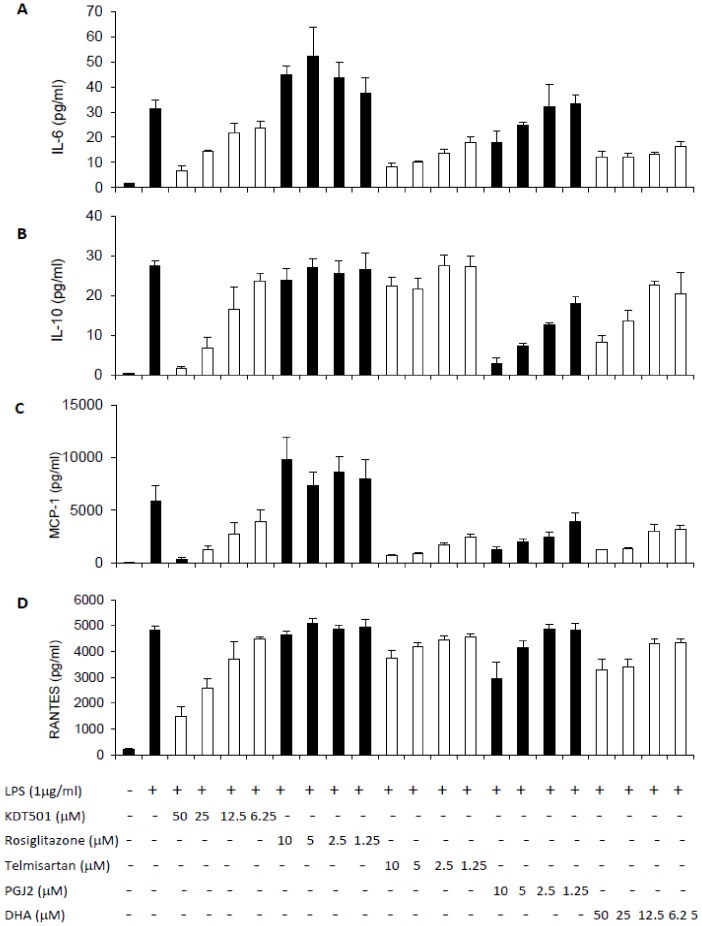
KDT501 reduced TNF-α and LPS-mediated inflammatory markers in THP-1 monocytes. THP-1 cells were pre-incubated in the absence or in the presence of various concentrations of KDT501, rosiglitazone, telmisartan, DHA and PGJ2 for 1 hour, and then stimulated with LPS (1 µg/mL) overnight. MCP-1, IL-6, IL-10 and RANTES levels in the medium were quantified as described in Methods. Data represent the mean ± SD from 4 independent samples. * Mean values significantly different from LPS stimulation (p<0.05).

To further understand how KDT501 is differentiated from classical PPARγ agonists, we performed studies in monocytic THP-1 cells, an established inflammatory cell line that expresses PPARγ. Changes in cell viability were not observed for any of the compounds at the highest dose used. KDT501 (6.25 to 50 µM) dose-dependently reduced LPS stimulated inflammatory mediators MCP-1, RANTES and IL-6 (Figure 2). Telmisartan, DHA and 5-deoxy-Δ12,14- PGJ2 (PGJ2), a naturally occurring PPARγ ligand, also inhibited these cytokines in a concentration-dependent manner. On the other hand, rosiglitazone did not significantly affect any of the inflammatory markers in this study, providing an obvious distinction between KDT501 and classic TZD's, and suggesting that the anti-inflammatory properties of KDT501 are independent of PPARγ in this cell model.

### Effect of KDT501 on lipogenesis in adipocytes

**Figure 3:**
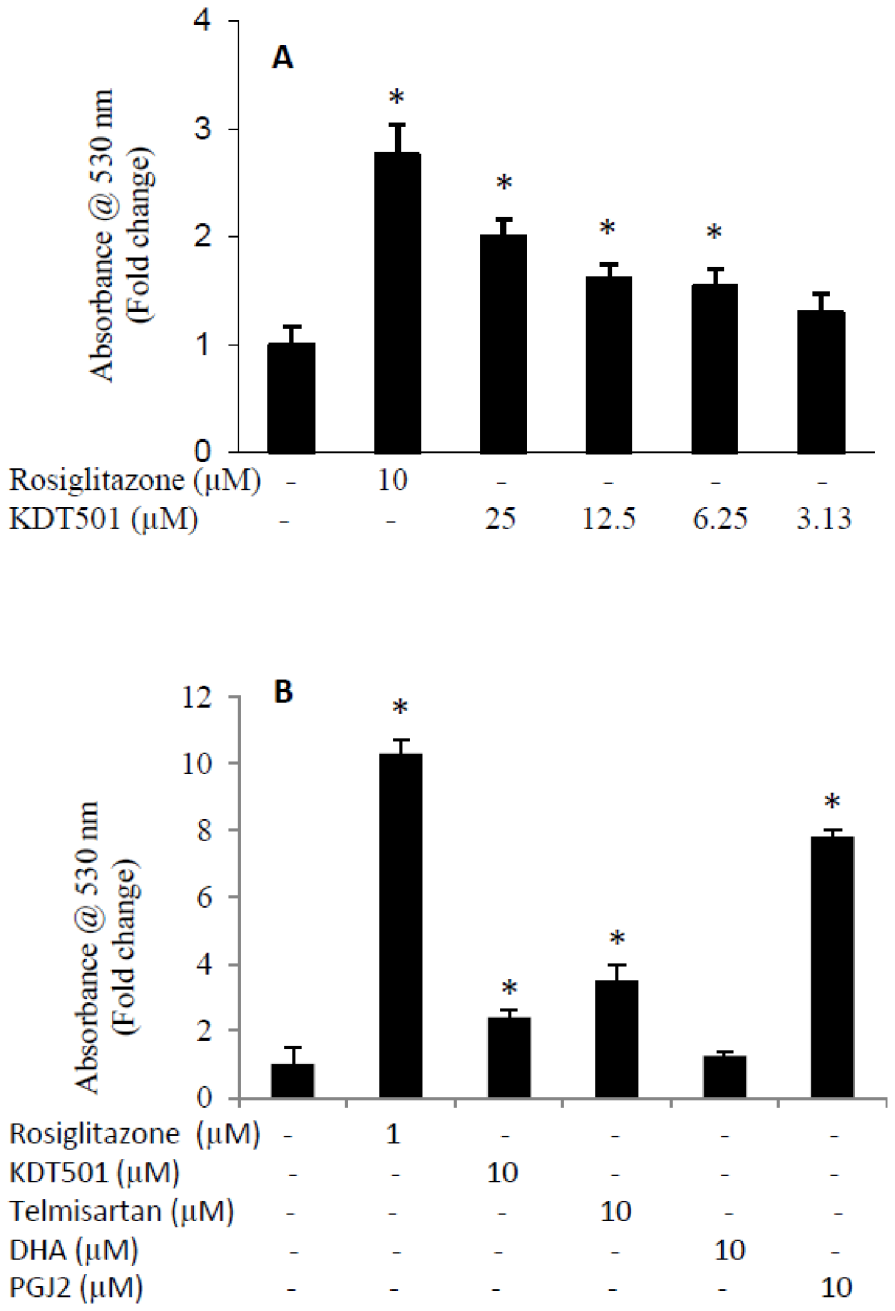
Effect of KDT501 on lipid accumulation in adipocytes. (A) 3T3L1 adipocytes were treated with rosiglitazone (10 uM) or various concentrations of KDT501 for 6 days. (B) Human subcutaneous adipocytes were treated with rosiglitazone (1 µM), KDT501 (10 µM), telmisartan (10 µM), DHA (10 µM) or PGJ2 (10 µM) for 10 days. Intracellular lipid was quantified with Oil Red O staining and the data represented as fold induction compared to DMSO negative control. Representative data expressed as mean ± SD from 4 independent samples. * Mean values significantly different from control (p<0.05).

Since PPARγ is known to be involved in adipogenesis, we evaluated the role of KDT501 in *de novo* lipogenesis in 3T3L1 adipocytes and human subcutaneous adipocytes. Rosiglitazone (10 µM) increased lipogenesis about 2.8-fold in 3T3L1 adipocytes. KDT501 dose-dependently (3.125 to 25 µM) increased lipogenesis with maximum activation of two-fold (Figure 3A). In human subcutaneous adipocytes, rosiglitazone (1 µM), PGJ2 (10 µM), telmisartan (10 µM) and KDT501 (10 µM) increased lipogenesis by 10.3, 8.8, 3.5 and 2.4-fold respectively (Figure 3B). These data are consistent with the observed PPARγ reporter activities for the compounds and demonstrate that KDT501 is a weak, partial PPARγ agonist. No significant changes in lipogenesis were observed with DHA.

### Gene expression in subcutaneous human adipocytes

Since adipocytes are one of the primary targets of classical PPARγ agonists such as rosiglitazone, we undertook a comparison of the gene expression profiles elicited by rosiglitazone and KDT501 in this cell type. The concentration necessary for maximum PPARγ activation and lipogenesis in cell-based assays was employed. For comparative purposes, we included telmisartan, which has previously been reported to exhibit weak PPARγ agonist activity [27].

**Figure 4:**
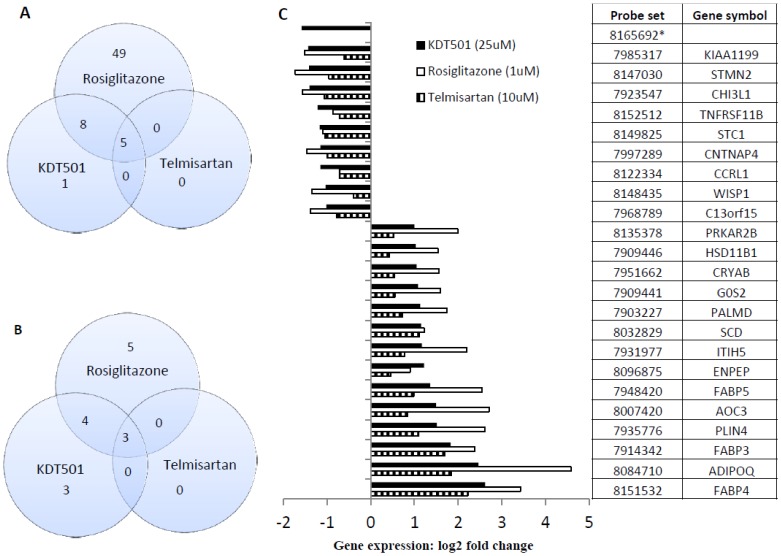
Human subcutaneous adipocyte gene signatures. Cells were treated with test compounds rosiglitazone (1 µM), telmisartan (10 µM) or KDT501 (25 µM) for 5 days. Gene arrays were performed and the probe sets selected were those with at least a 2-fold change as described in methods. Venn diagram represents up-regulated (A) and down regulated genes (B). KDT501-mediated (probe set 24) 3-way gene-intersection (rosiglitazone, KDT501 and telmisartan) were presented (C). * Probe set values for rosiglitazone and telmisartan are not qualified to include in the figure.

In our analysis, we considered only those transcripts that were changed by at least 2-fold, regardless of test article. The data summarized in the accompanying Venn diagram reveal clear differences between KDT501 and rosiglitazone (Figure 4). Of the 62 transcripts stimulated at least 2-fold by rosiglitazone, KDT501 had stimulatory effects on only 14, 5 of which were also stimulated by telmisartan (p<0.0001; Fisher's exact test). Similarly, KDT501 reduced 10 transcripts, of which only 7 were common to rosiglitazone. In addition, KDT501 changed 4 transcripts (3 up and 1 down), which were not observed in either rosiglitazone or telmisartan groups. Interestingly, telmisartan changed 8 (5 up and 3 down) transcripts, which were common to both rosiglitazone and KDT501. Gene ontology data for biological processes predict that both KDT501 and telmisartan influence brown fat cell differentiation (Table 1).

**Table 1:** Gene expression profile in human subcutaneous adipocytes.

Rank	Rosiglitazone (probe set 74)	P value	E value	Pop Hits	Enrich
1	regulation of cell-cell adhesion involved in gastrulation	4.70E-05	1.41E-04	3	167.0
2	regulation of heterotypic cell-cell adhesion	4.70E-05	1.41E-04	3	167.0
3	regulation of gastrulation	4.70E-05	1.41E-04	3	167.0
4	positive regulation of cholesterol storage	1.60E-04	8.00E-04	5	100.2
5	positive regulation of lipid storage	5.50E-04	4.95E-03	9	55.7
6	negative regulation of I-kappaB kinase/NF-kappaB cascade	5.50E-04	4.95E-03	9	55.7
7	regulation of cholesterol storage	8.40E-04	9.24E-03	11	45.5
8	regulation of embryonic development	1.20E-03	1.56E-02	13	38.5
9	positive regulation of foam cell differentiation	1.20E-03	1.56E-02	13	38.5
10	regulation of glutamate secretion	1.20E-03	1.56E-02	13	38.5
11	regulation of fatty acid biosynthetic process	1.60E-03	2.40E-02	15	33.4
12	negative regulation of endocytosis	1.60E-03	2.40E-02	15	33.4
13	response to vitamin D	1.80E-03	2.88E-02	16	31.3
14	lipid storage	2.30E-03	4.14E-02	18	27.8
15	social behavior	2.60E-03	4.94E-02	19	26.4
	**KDT501 (probe set 24)**				
1	response to hormone stimulus	1.50E-05	5.51E-03	367	10.5
2	response to endogenous stimulus	2.60E-05	1.05E-02	405	9.5
3	brown fat cell differentiation	6.20E-04	1.49E-02	24	53.7
4	response to steroid hormone stimulus	1.90E-04	3.65E-02	192	13.4
5	fatty acid metabolic process	2.20E-04	4.36E-02	198	13.0
	**Telmisartan (probe set 8)**				
1	brown fat cell differentiation	8.80E-03	1.08E-03	24	187.9
2	fat cell differentiation	1.90E-02	1.17E-02	53	85.1
3	fatty acid metabolic process	2.10E-03	1.19E-02	198	34.2
4	response to drug	2.50E-03	1.66E-02	216	31.3
5	regulation of inflammatory response	2.80E-02	3.50E-02	76	59.3
6	response to glucocorticoid stimulus	2.90E-02	3.74E-02	78	57.8
7	response to corticosteroid stimulus	3.10E-02	4.93E-02	85	53.1

Gene expression fold-change with test compounds larger than 1 log2 ratio (±1) over control with a p-value (Fisher exact) <0.05 and PADE value <15. The Bonferroni adjusted p-value (<0.05) was used for ranking and is shown as “E-Value”. The GO terms for KDT501 (probe set 24), rosiglitazone (probe set 74) and telmisartan (probe set 8) denote direct biological pathways.

### DIO mice

#### Body composition

**Figure 5:**
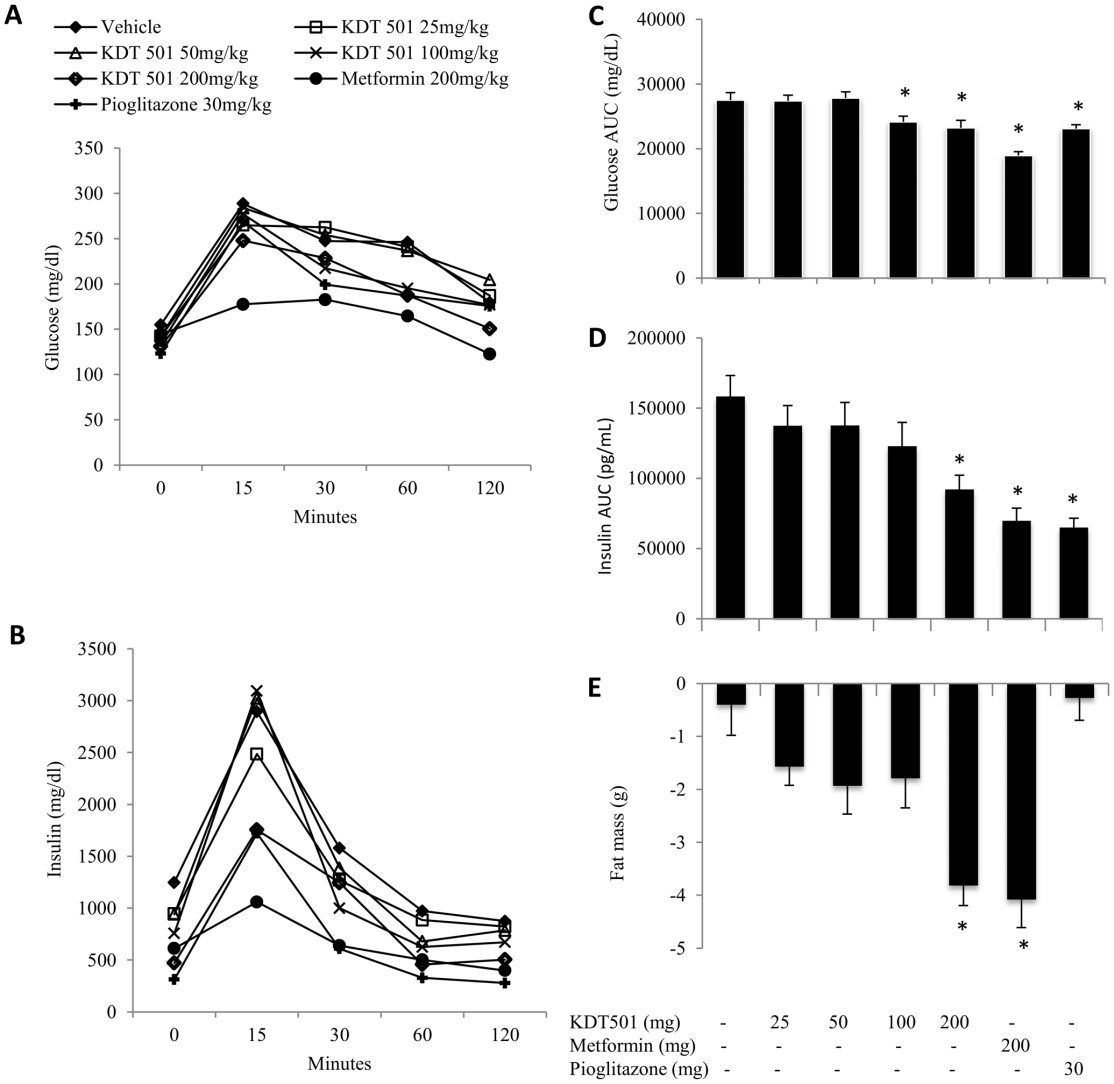
KDT501 reduces body fat and improves glucose metabolism. The test compounds were given orally twice daily for a month. Oral glucose tolerance test (OGTT) conducted as described in methods; glucose (A), insulin (B), glucose AUC (C) and insulin AUC (D) levels were presented. Body fat as determined by QNMR at the end of the study were presented (E). Data expressed as Mean ±SEM. * Mean values significantly different from vehicle control group (p<0.05).

Since KDT501 regulates inflammation and lipid metabolism in *in vitro* models, we evaluated KDT501 to determine its effect on glucose metabolism and weight gain in DIO mice. At the start of the experiment, the groups were well matched with regard to body weight and there were no significant differences seen in the body weight or percentage of body fat between the seven test groups. Interestingly, we also observed that the total weight loss is equivalent to fat loss (∼4 g) in the high dose KDT501 (200 mg/kg bid) group (data not shown). Significant reduction in fat mass was observed by the end of the study for KDT501 (doses ≥100 mg/kg bid) and metformin (Figure 5E).

*OGTT*: After four weeks on a high-fat diet, animals treated with metformin and pioglitazone exhibited significantly reduced glucose and insulin AUC's (Figures 5C and 5D) compared to controls. Mice treated with KDT501 (≥100 mg/ kg) also showed significant reduction in glucose AUC. The magnitude of reduction was comparable to that seen with 30 mg/kg pioglitazone. The effect on insulin was similar, with significant reductions in the AUC for metformin and pioglitazone as well as 200 mg/ kg KDT 501, while there are indications that insulin levels were reduced at lower doses of KDT 501.

### ZDF rats

#### Body weight gain

**Figure 6:**
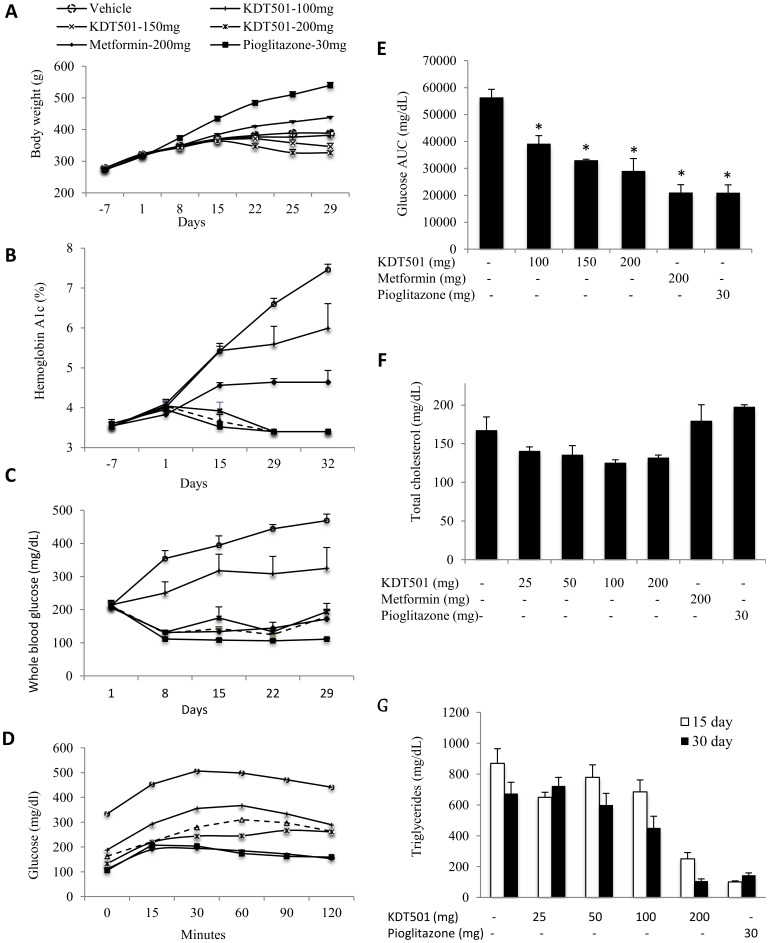
KDT501 reduces weight gain, improves glucose and fatty acid metabolism in ZDF rats. Vehicle control (black circles) and test compounds KDT501 (100 mg  =  white circles, 150 mg  =  white triangles, 200 mg  =  white squares), metformin (black diamonds) and rosiglitazone (black squares) were administered orally twice daily for up to 32 days. Weight gain (A), hemoglobin A1c (B) and whole blood glucose levels (C) were presented. At the end of the study, oral glucose tolerance tests (OGTT) were conducted as described in the methods; glucose (D) and glucose AUC levels (E) were presented. Total cholesterol levels (F) were measured in the plasma. Triglyceride levels were measured at day 15 and day 30 (G). Data expressed as Mean ±SEM. * Mean values significantly different from vehicle control group (p<0.05).

With the exception of groups receiving KDT501 (150 and 200 mg/kg), all animals gained weight throughout the trial (Figure 6A). Two weeks into the trial, animals receiving 200 mg/kg KDT501 began to lose weight, an effect observed one week later in the 150 mg/kg group (Figure 6A).

#### Effect on HbA1c

At the end of the study, 100 mg/kg of KDT501 reduced HbA1c equivalent to levels observed with metformin, while higher doses of KDT501 as well as pioglitazone reduced HbA1c levels to approximately half that of the vehicle-treated control (Figure 6B).

#### Non-fasting blood glucose

Administration of KDT501 (150 or 200 mg/kg) prevented the dramatic increase in blood sugar seen in vehicle-treated control animals as they progressed to frank diabetes (Figure 6C). KDT501 (200 mg/ kg) was equivalent in this regard to metformin and pioglitazone.

#### OGTT

The reduction seen in non-fasting blood glucose was mirrored when animals were subjected to an OGTT (Figure 6D), and all doses of KDT501 significantly reduced the AUC relative to the vehicle-treated control group (Figure 6E).

#### Effect on cholesterol and triglycerides

In another ZDF rat study, we evaluated the effect of KDT501 on total cholesterol and triglyceride levels in the plasma. KDT501 reduced cholesterol, but neither metformin nor pioglitazone had this effect (Figure 6F). Similar to pioglitazone, KDT501 reduced triglycerides (Figure 6G), with KDT501 (200 mg/kg) exhibiting the largest reduction by the end of the study.

## Discussion

In the present study, we evaluated the effect of KDT501 on several metabolic traits associated with insulin resistance and T2D. Oral administration of KDT501 in DIO mouse and ZDF rat models of diabetes improved glucose metabolism and reduced plasma HbA1c, an important biomarker in diabetes. KDT501 dose-dependently reduced weight gain and total cholesterol in ZDF rats, while rats treated with metformin or pioglitazone gained weight. KDT501 also reduced total cholesterol, unlike metformin and pioglitazone. These results suggest that the anti-diabetic mechanism of KDT501 differs from that of both metformin and pioglitazone and that KDT501 may be a novel therapeutic for the treatment of T2D and related conditions in humans.

In recent decades, several groups have reported that inflammation plays an important role in insulin resistance. We therefore evaluated KDT501 in several inflammatory models, including monocytic cells, THP-1 cells, RAW2647 macrophages, and human synovial fibroblasts from rheumatoid arthritis subjects (data not shown). KDT501 inhibited several inflammatory mediators (MCP-1, IL-6 and RANTES) in a dose-dependent manner in LPS-activated THP-1 cells (Figure 2). Although PPARγ has been reported to be present in this human monocytic cell line [28], an observation that we have confirmed, rosiglitazone failed to affect any of these inflammatory markers. In RAW264.7 cells, the induction of LPS-activated PGE_2_ and NO was reduced by KDT501, but not with rosiglitazone (results not shown). These data demonstrate that the anti-inflammatory mechanism of KDT501 differs from that of rosiglitazone and is independent of PPARγ expression, thus serving to highlight substantial differences between KDT501 and the glitazones. Several other natural compounds have been found to exhibit PPARγ-dependent (e.g., resveratrol) and independent (e.g., luteolin) anti-inflammatory properties [29] in a co-culture system of adipocytes and macrophages. These studies, in combination with our findings, suggest that the anti-inflammatory activity of many partial/ non-TZD PPARγ agonists is mediated independently of PPARγ activation.

The incidence of diabetes is associated with obesity, a chronic medical condition with increasing worldwide prevalence. Many studies indicate that inflammation plays an important role in diet-induced obesity and diabetes. It has been shown that inflammatory markers such as MCP-1 are involved in the recruitment of inflammatory macrophages to adipose tissue, contributing to the development of insulin resistance and progressing, in many cases, to T2D [30], [31]. MCP-1 deficiency increases M2 macrophages and ameliorates insulin sensitivity and fatty liver in diabetic mice [32]. In obese adipose tissue, 40% of all cells are infiltrating macrophages [3]. Results of the NIH-sponsored TINSAL-T2D clinical studies have demonstrated that salsalate, a non-steroidal anti-inflammatory drug, lowers HbA1c, fasting glucose and markers of inflammation in T2D patients [33].

The pleiotropic effects of many natural compounds (e.g., resveratrol, curcumin, EGCG) are well documented. KDT501 appears to be involved in the regulation of multiple insulin-sensitizing mechanisms, including the reduction of inflammation and activation of lipogenesis. In Cerep BioPrint assays, we identified only three biological targets, suggesting both an unusual specificity as well as a good safety profile for KDT501. KDT501 activates transcriptional activity of one of these targets, PPARγ, but not of PPARα or PPARδ, indicating ligand-binding specificity. Specificity towards PPARγ suggests reduced side effects associated with nonspecific PPAR agonists, such as PPARα-mediated hepatocarcinoma. Recent reports show that pharmacological activation of a second KDT501 target, AGTR2, in combination with PPARγ activation, ameliorates insulin resistance and reverses β-cell damage in diabetic pancreatic tissue in T2D mice [34]. AT1 receptor blockers are known to improve insulin resistance and delay the onset of diabetes [35]. AT1 receptor-mediated anti-diabetic effects are thought to be mediated through the activation of AT2 receptors by unbound angiotensin [36]. Further research is required to understand the full impact of KDT501's PPARγ and AGTR2-mediated mechanisms and their functional roles in diabetes.

Recently, it has been reported [37] that selective PPAR modulators display a selective gene regulatory profile in comparison with the full PPARγ agonist rosiglitazone. Our comparison of the gene expression profiles of human subcutaneous adipocytes treated with KDT501, rosiglitazone, and the weak PPARγ agonist telmisartan revealed that KDT501 produced not only an attenuated gene signature compared to rosiglitazone, but also regulated a different set of genes than those modulated by rosiglitazone or telmisartan. We found that saturated concentrations of telmisartan also produced an attenuated gene expression profile, compared to rosiglitazone. These data are consistent with other published gene expression profiles where partial agonists had a similar pattern of attenuated gene expression compared to full agonists [37]. Consistent with lipid accumulation data in adipocytes (Fig. 3), we observed the activation of genes involved in lipid metabolism (perilipin, fatty acid binding proteins 3, 4 & 5 etc.). We also observed the induction of adiponectin, a known PPARγ-dependent, anti-inflammatory gene in adipocytes. Interestingly, KDT501 inhibits chemokine receptor gene (CCLR1), which is known to bind MCP-1, an important inflammatory mediator that attracts macrophages to adipocytes. Gene ontology data (Table 1) suggest that rosiglitazone (probe set 77), KDT501 (probe set 24) and telmisartan (probe set 8) are involved in common (fat metabolism) as well as independent biological processes. Gene ontology data also indicate that both KDT501 and telmisartan may influence brown fat cell differentiation, which is important in thermogenesis. Additional studies are required to confirm the role of KDT501 in the regulation of brown fat physiology and thermoregulation.

Our studies show that the novel compound KDT501 improves blood glucose levels and insulin resistance in rodent models of T2D. We have also demonstrated that KDT501 moderates inflammation and adipose tissue function through a unique mechanism that is complementary and distinct from current anti-diabetic drugs. Additional research is needed to further delineate this molecule's pleiotropic mechanism and potential as a next-generation therapeutic for the treatment of human metabolic disorders.

## References

[1] Hotamisligil GS, Shargill NS, Spiegelman BM (1993) Adipose expression of tumor necrosis factor-alpha: direct role in obesity-linked insulin resistance. Science 259: 87–91. 7678183 10.1126/science.7678183

[2] Siriwardhana N, Kalupahana NS, Cekanova M, Lemieux M, Greer B, et al (2013) Modulation of adipose tissue inflammation by bioactive food compounds. J Nutr Biochem 24: 613–623. 23498665 10.1016/j.jnutbio.2012.12.013

[3] Weisberg SP, McCann D, Desai M, Rosenbaum M, Leibel RL, et al (2003) Obesity is associated with macrophage accumulation in adipose tissue. J Clin Invest 112: 1796–1808. 14679176 10.1172/JCI19246 PMC296995

[4] Ferrante AW Jr (2007) Obesity-induced inflammation: a metabolic dialogue in the language of inflammation. J Intern Med 262: 408–414. 17875176 10.1111/j.1365-2796.2007.01852.x

[5] Lumeng CN, DelProposto JB, Westcott DJ, Saltiel AR (2008) Phenotypic switching of adipose tissue macrophages with obesity is generated by spatiotemporal differences in macrophage subtypes. Diabetes 57: 3239–3246. 18829989 10.2337/db08-0872 PMC2584129

[6] Odegaard JI, Ricardo-Gonzalez RR, Goforth MH, Morel CR, Subramanian V, et al (2007) Macrophage-specific PPARgamma controls alternative activation and improves insulin resistance. Nature 447: 1116–1120. 17515919 10.1038/nature05894 PMC2587297

[7] Brun RP, Spiegelman BM (1997) PPAR gamma and the molecular control of adipogenesis. J Endocrinol 155: 217–218. 9415052 10.1677/joe.0.1550217

[8] Peraza MA, Burdick AD, Marin HE, Gonzalez FJ, Peters JM (2006) The toxicology of ligands for peroxisome proliferator-activated receptors (PPAR). Toxicol Sci 90: 269–295. 16322072 10.1093/toxsci/kfj062

[9] Nesto RW, Bell D, Bonow RO, Fonseca V, Grundy SM, et al (2003) Thiazolidinedione use, fluid retention, and congestive heart failure: a consensus statement from the American Heart Association and American Diabetes Association. October 7, 2003. Circulation 108: 2941–2948. 14662691 10.1161/01.CIR.0000103683.99399.7E

[10] Berger JP, Petro AE, Macnaul KL, Kelly LJ, Zhang BB, et al (2003) Distinct properties and advantages of a novel peroxisome proliferator-activated protein [gamma] selective modulator. Mol Endocrinol 17: 662–676. 12554792 10.1210/me.2002-0217

[11] Acton JJ 3rd, Black RM, Jones AB, Moller DE, Colwell L, et al (2005) Benzoyl 2-methyl indoles as selective PPARgamma modulators. Bioorg Med Chem Lett 15: 357–362. 15603954 10.1016/j.bmcl.2004.10.068

[12] Chang CH, McNamara LA, Wu MS, Muise ES, Tan Y, et al (2008) A novel selective peroxisome proliferator-activator receptor-gamma modulator-SPPARgammaM5 improves insulin sensitivity with diminished adverse cardiovascular effects. Eur J Pharmacol 584: 192–201. 18346728 10.1016/j.ejphar.2007.12.036

[13] Motani A, Wang Z, Weiszmann J, McGee LR, Lee G, et al (2009) INT131: a selective modulator of PPAR gamma. J Mol Biol 386: 1301–1311. 19452630 10.1016/j.jmb.2009.01.025

[14] Acton JJ 3rd, Akiyama TE, Chang CH, Colwell L, Debenham S, et al (2009) Discovery of (2R)-2-(3-{3-[(4-Methoxyphenyl)carbonyl]-2-methyl-6-(trifluoromethoxy)-1H-indol-1 -yl}phenoxy)butanoic acid (MK-0533): a novel selective peroxisome proliferator-activated receptor gamma modulator for the treatment of type 2 diabetes mellitus with a reduced potential to increase plasma and extracellular fluid volume. J Med Chem 52: 3846–3854. 19507861 10.1021/jm900097m

[15] Guasch L, Sala E, Castell-Auvi A, Cedo L, Liedl KR, et al (2012) Identification of PPARgamma Partial Agonists of Natural Origin (I): Development of a Virtual Screening Procedure and In Vitro Validation. PLoS One 7: e50816. 23226391 10.1371/journal.pone.0050816 PMC3511273

[16] Castrillo A, Mojena M, Hortelano S, Bosca L (2001) Peroxisome proliferator-activated receptor-gamma-independent inhibition of macrophage activation by the non-thiazolidinedione agonist L-796,449. Comparison with the effects of 15-deoxy-delta(12,14)-prostaglandin J(2). J Biol Chem 276: 34082–34088. 11438523 10.1074/jbc.M102472200

[17] Bassaganya-Riera J, Guri AJ, Lu P, Climent M, Carbo A, et al (2011) Abscisic acid regulates inflammation via ligand-binding domain-independent activation of peroxisome proliferator-activated receptor gamma. J Biol Chem 286: 2504–2516. 21088297 10.1074/jbc.M110.160077 PMC3024745

[18] Guri AJ, Hontecillas R, Si H, Liu D, Bassaganya-Riera J (2007) Dietary abscisic acid ameliorates glucose tolerance and obesity-related inflammation in db/db mice fed high-fat diets. Clin Nutr 26: 107–116. 17000034 10.1016/j.clnu.2006.07.008

[19] Desai A, Konda VR, Darland G, Austin M, Prabhu KS, et al (2009) META060 inhibits multiple kinases in the NF-kappaB pathway and suppresses LPS–mediated inflammation in vitro and ex vivo. Inflamm Res 58: 229–234. 19169645 10.1007/s00011-008-8162-y

[20] Desai A, Darland G, Bland JS, Tripp ML, Konda VR (2012) META060 attenuates TNF-alpha-activated inflammation, endothelial-monocyte interactions, and matrix metalloproteinase-9 expression, and inhibits NF-kappaB and AP-1 in THP-1 monocytes. Atherosclerosis 223: 130–136. 22658256 10.1016/j.atherosclerosis.2012.05.004

[21] Konda VR, Desai A, Darland G, Bland JS, Tripp ML (2010) META060 inhibits osteoclastogenesis and matrix metalloproteinases in vitro and reduces bone and cartilage degradation in a mouse model of rheumatoid arthritis. Arthritis Rheum 62: 1683–1692. 20201075 10.1002/art.27441

[22] Vroegrijk IO, van Diepen JA, van den Berg SA, Romijn JA, Havekes LM, et al (2013) META060 protects against diet-induced obesity and insulin resistance in a high-fat-diet fed mouse. Nutrition 29: 276–283. 22985971 10.1016/j.nut.2012.05.004

[23] Yajima H, Ikeshima E, Shiraki M, Kanaya T, Fujiwara D, et al (2004) Isohumulones, bitter acids derived from hops, activate both peroxisome proliferator-activated receptor alpha and gamma and reduce insulin resistance. J Biol Chem 279: 33456–33462. 15178687 10.1074/jbc.M403456200

[24] Everard A, Geurts L, Van Roye M, Delzenne NM, Cani PD (2012) Tetrahydro iso-alpha acids from hops improve glucose homeostasis and reduce body weight gain and metabolic endotoxemia in high-fat diet-fed mice. PLoS One 7: e33858. 22470484 10.1371/journal.pone.0033858 PMC3314685

[25] Urban J, Dahlberg CJ, Carroll BJ, Kaminsky W (2013) Absolute Configuration of Beer's Bitter Compounds. Angew Chem Int Ed Engl 52: 1553–1555. 23239507 10.1002/anie.201208450 PMC3563212

[26] Babish JG, Pacioretty LM, Bland JS, Minich DM, Hu J, et al (2010) Antidiabetic screening of commercial botanical products in 3T3-L1 adipocytes and db/db mice. J Med Food 13: 535–547. 20521979 10.1089/jmf.2009.0110

[27] Benson SC, Pershadsingh HA, Ho CI, Chittiboyina A, Desai P, et al (2004) Identification of telmisartan as a unique angiotensin II receptor antagonist with selective PPARgamma-modulating activity. Hypertension 43: 993–1002. 15007034 10.1161/01.HYP.0000123072.34629.57

[28] Pang T, Benicky J, Wang J, Orecna M, Sanchez-Lemus E, et al (2012) Telmisartan ameliorates lipopolysaccharide-induced innate immune response through peroxisome proliferator-activated receptor-gamma activation in human monocytes. J Hypertens 30: 87–96. 22124178 10.1097/HJH.0b013e32834dde5f PMC3237779

[29] Hirai S, Takahashi N, Goto T, Lin S, Uemura T, et al (2010) Functional food targeting the regulation of obesity-induced inflammatory responses and pathologies. Mediators Inflamm 2010: 367838. 20508825 10.1155/2010/367838 PMC2876247

[30] Kanda H, Tateya S, Tamori Y, Kotani K, Hiasa K, et al (2006) MCP-1 contributes to macrophage infiltration into adipose tissue, insulin resistance, and hepatic steatosis in obesity. J Clin Invest 116: 1494–1505. 16691291 10.1172/JCI26498 PMC1459069

[31] Weisberg SP, Hunter D, Huber R, Lemieux J, Slaymaker S, et al (2006) CCR2 modulates inflammatory and metabolic effects of high-fat feeding. J Clin Invest 116: 115–124. 16341265 10.1172/JCI24335 PMC1307559

[32] Nio Y, Yamauchi T, Iwabu M, Okada-Iwabu M, Funata M, et al (2012) Monocyte chemoattractant protein-1 (MCP-1) deficiency enhances alternatively activated M2 macrophages and ameliorates insulin resistance and fatty liver in lipoatrophic diabetic A-ZIP transgenic mice. Diabetologia 55: 3350–3358. 22983634 10.1007/s00125-012-2710-2

[33] Goldfine AB, Fonseca V, Jablonski KA, Pyle L, Staten MA, et al (2010) The effects of salsalate on glycemic control in patients with type 2 diabetes: a randomized trial. Ann Intern Med 152: 346–357. 20231565 10.1059/0003-4819-152-6-201003160-00004 PMC3138470

[34] Ohshima K, Mogi M, Jing F, Iwanami J, Tsukuda K, et al (2012) Direct angiotensin II type 2 receptor stimulation ameliorates insulin resistance in type 2 diabetes mice with PPARgamma activation. PLoS One 7: e48387. 23155382 10.1371/journal.pone.0048387 PMC3498306

[35] Olivares-Reyes JA, Arellano-Plancarte A, Castillo-Hernandez JR (2009) Angiotensin II and the development of insulin resistance: implications for diabetes. Mol Cell Endocrinol 302: 128–139. 19150387 10.1016/j.mce.2008.12.011

[36] de Gasparo M, Catt KJ, Inagami T, Wright JW, Unger T (2000) International union of pharmacology. XXIII. The angiotensin II receptors. Pharmacol Rev 52: 415–472. 10977869

[37] Tan Y, Muise ES, Dai H, Raubertas R, Wong KK, et al (2012) Novel transcriptome profiling analyses demonstrate that selective peroxisome proliferator-activated receptor gamma (PPARgamma) modulators display attenuated and selective gene regulatory activity in comparison with PPARgamma full agonists. Mol Pharmacol 82: 68–79. 22496518 10.1124/mol.111.076679

